# Sigma-1 Receptor in Retina: Neuroprotective Effects and Potential Mechanisms

**DOI:** 10.3390/ijms23147572

**Published:** 2022-07-08

**Authors:** Zifen Xu, Yushuang Lei, Huan Qin, Shiyao Zhang, Ping Li, Kai Yao

**Affiliations:** Institute of Visual Neuroscience and Stem Cell Engineering, College of Life Sciences and Health, Wuhan University of Science and Technology, Wuhan 430065, China; xuzifen92@outlook.com (Z.X.); leiyushuang79@outlook.com (Y.L.); qhainne2021@outlook.com (H.Q.); shiyaozhang0525@outlook.com (S.Z.); amy4115@163.cm (P.L.)

**Keywords:** sigma-1 receptor, degenerative retinal diseases, neuroprotection, Nrf2 signaling pathway, MAPK signaling pathway

## Abstract

Retinal degenerative diseases are the major factors leading to severe visual impairment and even irreversible blindness worldwide. The therapeutic approach for retinal degenerative diseases is one extremely urgent and hot spot in science research. The sigma-1 receptor is a novel, multifunctional ligand-mediated molecular chaperone residing in endoplasmic reticulum (ER) membranes and the ER-associated mitochondrial membrane (ER-MAM); it is widely distributed in numerous organs and tissues of various species, providing protective effects on a variety of degenerative diseases. Over three decades, considerable research has manifested the neuroprotective function of sigma-1 receptor in the retina and has attempted to explore the molecular mechanism of action. In the present review, we will discuss neuroprotective effects of the sigma-1 receptor in retinal degenerative diseases, mainly in aspects of the following: the localization in different types of retinal neurons, the interactions of sigma-1 receptors with other molecules, the correlated signaling pathways, the influence of sigma-1 receptors to cellular functions, and the potential therapeutic effects on retinal degenerative diseases.

## 1. Introduction

The sigma receptor was once mistaken for one subtype of opioid receptor that consists of three subtypes: kappa, mu, and sigma [1]. Subsequently, it was found that the sigma receptor was distinguished from universal opioid receptors since the prototypical opioid receptor antagonists, naloxone and etorphine, failed to inhibit the effect of sigma ligand SKF10047. More specifically, the levorotatory isomer of SKF10047 acts as an opioid receptor antagonist, in contrast, dextroisomer has an agonist effect to sigma receptor [2,3,4,5].

Sigma receptors were classified as sigma-1 and sigma-2 subtypes, based on their distinct binding site and molecular mass [6,7]. The human sigma-1 receptor gene sequence is seated on chromosome 9, band p13 [8]. Sigma-1 receptor protein was first cloned in guinea pig liver tissue with a 1857bp open reading frame in length coding for a 223 amino acid sequence, and the molecular mass of protein is 25.3kD [9]. Successively, sigma-1 receptor was cloned in human [10] and rat brains [11]. The cloned amino acid sequences of the mammalian sigma-1 receptor shared a large degree homologous in guinea pig liver, a human placenta, and rat brain, whereas the protein shared no discernible homology with any other mammalian proteins [9,12]. It was interesting to note that the sigma-1 receptor not only showed a prominent amino acid sequence homologous with the yeast sterol C8–C7 isomerase (ERG protein), but also implied pharmacological similarities between sigma-1 receptor binding site and fungi sterol isomerase [13]. 

As the research moves forward, the sigma-1 receptor has been found to play an important role in both physiological and pathological conditions. The initial studies postulated that the protein contained only one transmembrane domain [9,10,14,15]; nevertheless, and before long, the research of Ayder denoted it to be two membrane-spanning segments and two cytoplasmic terminals [16]. Finally, crystallographic studies discovered that the human sigma-1 receptor was a trimeric structure encompassing one-pass transmembrane domain in each protomer. The domain comprised of a β-barrel fold flanked by four α helices. Biochemical analysis showed that the ligand binding site was seated in the center of a β-barrel fold [17], which was different from the previous one single and twice transmembrane architecture [9,16]. Subsequently, Schmidt and his colleagues further revealed the molecular basis for ligand recognition of the sigma-1 receptor. In general, ligands, both agonists and antagonists of the sigma-1 receptor, interacted electrostatically with the sigma-1 receptor positively charged nitrogen. The main difference between them was that the antagonist occupied a more linear binding pocket, while the agonist adopted a nonlinear binding site [18].

**Figure 1:**
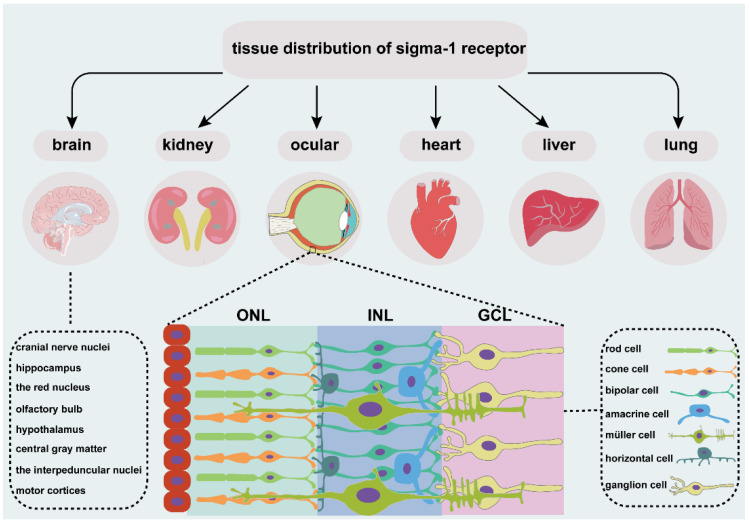
An overview of tissue distribution of sigma-1 receptor. It is located in different organs, such as brain, kidney, heart, liver, lung, and ocular. Sigma-1 receptor expressed in different cell types of retina, including photoreceptor cell, retinal interneuron (bipolar cell, amacrine cell, and horizontal cell), Müller cell and retinal ganglion cell. ONL, outer nuclear layer; INL, the inner nuclear layer; GCL, ganglion cell layer.

The sigma-1 receptor is widely expressed in several organs (Figure 1), such as endocrine, immune, and reproductive tissues, the liver, kidney, lung, and heart, and the central and peripheral nervous systems [19,20,21,22,23,24,25,26,27]. Early investigations focused more on the expression of sigma-1 receptors in the nervous system. In the central nervous system, the sigma-1 receptor is localized in various cranial nerve nuclei, the hippocampus, the red nucleus, the interpeduncular nucleus, and the mid-layers of the primary and secondary motor cortices [25], the olfactory bulb, the hypothalamus, the central gray matter lateral to the aqueduct, the medulla, the small and medium neuron of the superficial layer in the spinal cord gray matter [19], and the ventral motoneurons of the spinal cord [24]. The sigma-1 receptor is also distributed in astrocytes of the spinal cord [19], the dorsal root ganglion [20,23], the Schwann cells of a rat sciatic nerve [21]], and the oligodendrocytes in rats [22]. The sigma-1 receptor normally forms complexes with its binding protein (Bip) targeted on ER-MAM [28] and is mainly located in ER-MAM [29], the plasma membrane [20,30]], and the nuclear envelope [20,31]. The sigma-1 receptor transforms from ER-MAM to plasma and the nuclear membrane to perform the function through interactions with its ligands [28].

## 2. Expression and Molecular Interactions of Sigma-1 Receptor in Retina

### 2.1. Expression of Sigma-1 Receptor in Retinal Neurons

The vertebrate retina is composed of three cell nuclear layers (Figure 1), respectively, the outer nuclear layer (ONL), the inner nuclear layer (INL), and the ganglion cell layer (GCL) and two plexiform layers, the inner plexiform layer (IPL) and the outer plexiform layer (OPL) [32]. Light-sensitive cells, rods and cones, which are responsible for scotopic and photopic vision, are located in the ONL. The INL consists of Müller glial cells and three main interneurons, bipolar cells, horizontal cells, and amacrine cells. They transmit the optical signals from the ONL to the GCL. Finally, optic ganglion cells transmit the preliminarily integrated information to visual processing areas in the brain through optic nerves [33] to retinal synaptic terminals [34].

**Figure 2:**
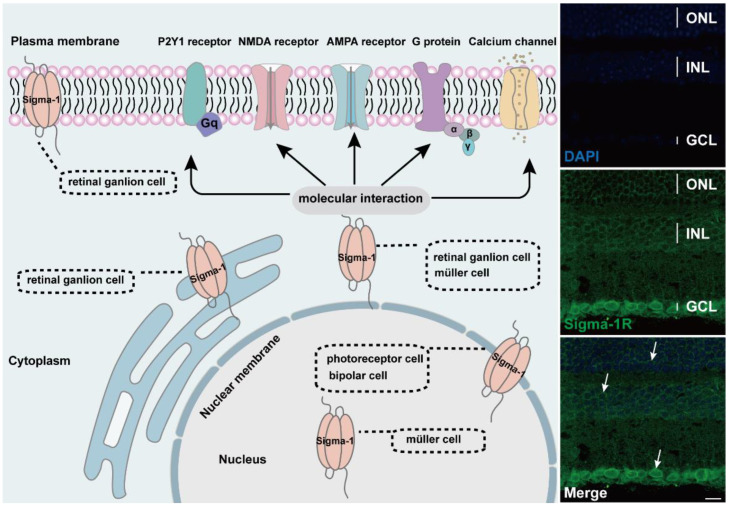
Diagram of subcellular distribution and molecular interaction of sigma-1 receptors in retina. The expression of sigma-1 receptors in mouse retina by immunofluorescence was shown on the right side of schematic. Confocal scanning showed that sigma-1 receptor mainly expressed in nuclear membrane in ONL, INL, and GCL and the signals were labeled with white arrows.

Photoreceptor cells are the initiators in visual signal transduction. Cell dysfunction and the death of photoreceptor cells drive visual impairment and even blindness. Studies have shown that the sigma-1 receptor is closely related to the function of photoreceptor cells. The expression of the sigma-1 receptor in the retina occurs in the early developmental stage. As showed from the IHC analysis, the sigma-1 receptor started to express as early as a day 16 embryo (E16) and increased progressively with the maturation of the retina from postnatal day1(p1) to p10 and kept stable at p30 in mice retina [34]. The 661W cell is a retinal photoreceptor cell line, which stably expresses blue and green cone pigments and cone arrestin. Immunoblotting detected the protein of the sigma-1 receptor in the whole cell lysates of 661W. Further analysis showed its expression in the cytoplasm, nucleus, and even the nuclear membrane. In wild type mice, the sigma-1 receptor was distributed in the nuclei of the cone and rod cells [35,36,37] (Figure 2). Similar results were detected in heterozygous and homozygous mice [38,39]. In situ hybridization analysis showed the mRNA of the sigma-1 receptor in the mouse inner segment photoreceptor cell [40,41]. Electron microscopic observation of photoreceptor cells showed the expression in the synaptic terminal of bovine retina, while nothing but in nuclear membranes in mouse retina. In these two species, the subcellular expression of the sigma-1 receptor was quite different in photoreceptor cells [34].

Retinal ganglion cells integrate visual information from retinal interneurons and project to the visual cortex, although they are a very minor portion in whole retinal neurons. Retinal ganglion cells are tightly connected to energy metabolism, cellular stress, and axonal transporting. The death of retinal ganglion cells was associated with glaucoma [42]. Substantial in vitro and in vivo studies have demonstrated that the sigma-1 receptor is ubiquitously expressed on retinal ganglion cells. The protein and mRNA of the sigma-1 receptor was expressed in different mouse strains and animal models of retinal disease. Western blot and immunofluorescent staining detected signals of the sigma-1 receptor in cultured primary ganglion cells of rats and mice [43,44,45,46]. The sigma-1 receptor was localized in retinal ganglion cells of albino ICR mice by in situ hybridization analysis and immunolabeling [40] The sigma-1 receptor was also present in the retinal ganglion cells of streptozotocin-induced diabetes mice [47] and *Ins2^Akita/+^* diabetes mice [48,49]. However, the expression level of the sigma-1 receptor was decreased in heterozygous mice and there was no signal in the sigma-1 receptor knockout mice [38]. The subcellular distribution of the sigma-1 receptor was mainly located in the cell membrane and the cytoplasm [39]. Similarly, a retrograde tracking experiment indicated the colocalization of sigma-1 receptors in the rat retinal cell membrane and the cytoplasm of ganglion cells [50]. Further study found the subcellular distribution in the nuclear membrane as well as the pool connecting the nuclear membrane and the endoplasmic reticulum membrane, whereas there were no signals in dendrites [34]. One earlier report showed the distribution in optic nerve head [40].

Müller cells are the major glial cell type spanning all retinal layers and normally contribute to retinal structure and homeostasis [33]. An early RT-PCR analysis in the rat Müller cell line discovered mRNA expression of the sigma-1 receptor, and in situ hybridization performed in mouse retina showed slight but detectable mRNA signals in Müller cells [40]. Subsequently, RT-PCR and immunoblotting analysis showed the expression of the sigma-1 receptor in primary mouse Müller cells (1MC) and rat Müller cell lines (rMC-1) and the co-localization with both nuclear membrane markers Lamin-A and endoplasmic reticulum membranes marker PDI, respectively [51]. The sigma-1 receptor was expressed in Müller glial cell in wide type mice [52].

Horizontal cells, bipolar cells, and amacrine cells are three predominant interneurons in retina. They receive visual input from photoreceptor cells and convey excitatory or inhibitory outputs to retinal ganglion cells [53]. Studies have reported the distribution of the sigma-1 receptor in the three mentioned retinal cells in retinal sections in detail. The sigma-1 receptor was co-localized with a specific horizontal cell marker, calbindin, in the cell body in the OPL. Amacrine cells compose a variety of cell subtypes, including GABAergic, dopaminergic, cholinergic, and glycinergic. Most of the GABAergic amacrine cells are co-expressed with sigma-1 receptors in the GCL. The dopaminergic marker of TH had a strong co-localization with sigma-1 receptors mainly in the INL cell body and dendrites, whereas the cholinergic marker of CHAT had a weak co-localization with the sigma-1 receptor in the INL. Part of the glycinergic cells were co-labeled with sigma-1 receptors in INL [50]. Nevertheless, the results of the EM observation revealed the presence of the sigma-1 receptor in the inner and outer membrane of nuclear, as well as a minor expression on ER in mouse retinal bipolar cells [34]. The sigma-1 receptor was also distributed in the cultured optic nerve head astrocytes (ONHAs), which were the most essential glial cells in the optic nerve head and pivotal for RGCs survival [54].

Apart from the expression in the retina, the sigma-1 receptor was also distributed in other ocular structures of various species and in other ocular cell lines associated with physiological and pathological functions. The earliest study concerning ocular tissue expressing sigma-1 receptors were the lacrimal glands [55]. Since then, a series of studies related to the expression in other ocular tissues were reported in succession, such as the iris and ciliary body, the RPE-choroid complex, the cornea, and the lens. The ocular cells expressing sigma-1 receptors included the columnar cells of the stratified epithelium, the corneal epithelial cells, the RPE cells, and the human lens epithelial cell line (FHL124). These varieties included bovine, rabbit, mouse, pig, cow, monkey, rat, and the human [15,34,38,40,56,57,58,59].

### 2.2. Molecular Interaction of Sigma-1 Receptor in Retina

The sigma-1 receptor acts as a novel molecular chaperone that regulates several cellular functions by the interaction with ion channels, receptors, kinases, and even RNA (Figure 2). (+) SKF10047, one agonist of the sigma-1 receptor, inhibited glutamate-induced intracellular Ca^2+^ influx in RGC-5 cells by fura-2AM intracellular calcium imaging [60]. In another report, the activation of the sigma-1 receptor reduced calcium concentrations by voltage-dependent L-type calcium gated channels in RGC cells [61]. In the meanwhile, the sigma-1 receptor decreased the NMDA-mediated excitatory postsynaptic potential of retinal ganglion cells by modulating the NMDA receptor, and the inhibition on the current amplitude was reversed by GDP-β, an inhibitor of the G protein coupled receptor [62,63]. (+) SKF10047 also suppressed the amplitude of light-evoked excitatory postsynaptic current (L-EPSC) activated by the AMPA receptor in rat retinal ON-ganglion cells. Similarly, the action of (+) SKF10047 was blocked by GDP-β-S. The possible mechanism was corelated to PKG activity regulating intracellular Ca^2+^ release [64], suggesting the possible interaction of the sigma-1 receptor with G-protein. Sigma-1 receptors regulated the metabolic glutamate receptor and purinergic P2Y1 receptor to alleviate the osmotic swelling of rat Müller cells upon hypoosmotic conditions [65]. Sigma-1 receptors also reduced LPS-induced inflammatory responses via regulating phosphorylation levels of extracellular signal-regulated kinase (ERK1/2) and C-JNK in retinal microglial [66]. An additional study reported that sigma-1 receptors decreased the NMDA-induced neurotoxic effect in RGCs through mediating ERK1/2 [67]. When cultured RGCs were deprived of oxygen and glucose, a mimic ischemic injury model, a sigma-1 receptor agonist upregulated the diminished level of phosphorylated ERK1/2, which was reversed by the antagonist BD1047 [44]. MiFinder miScript miRNA PCR array analysis showed that the expression of miR-214-3p, which was closely related to the oxidative stress response, was fivefold in *rd10/sigR^−/−^* mice compared with *rd10* mice. A (+)-PTZ intervention decreased miR-214-3p to about twofold. MiRNA targeted predictive program analysis revealed that the sigma-1sigma-1 receptor 3′UTR had a binding site for MMU-Mir-214-3p; it was the miR214-3p sequence GGACGAC, which can bind to the CCUGCUG sequence of the sigma-1 receptor [68]. Under higher oxidative stress conditions in the Müller cells, the sigma-1 receptor interacted with Nrf2 and cystine/glutamate exchange transporter (system x_c_^−^) to improve the level of antioxidant capacity [69].

## 3. Relevant Signaling Pathways of Sigma-1 Receptor in Retina

### 3.1. Nrf2 Signaling Pathway

**Figure 3:**
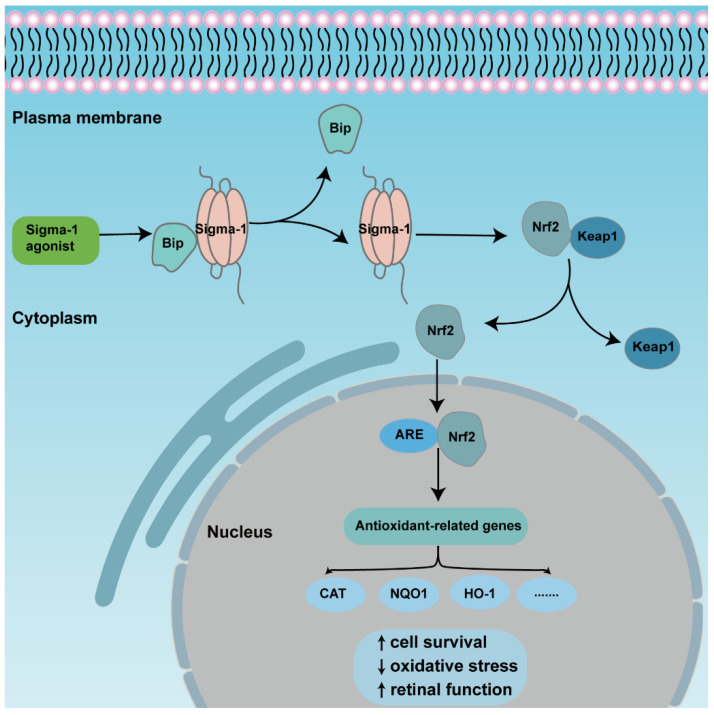
An overview of the mechanism of sigma-1 receptor modulates anti-oxidative function in retina through Nrf2 signaling pathway. The activation of sigma-1 receptor enhances releasing of Nrf2 from combined protein Keap1 and transforms from cytoplasm to nuclei. Nrf2 binds to ARE, then upregulates expression of downstream antioxidant genes., finally promoting cell survival and retinal function.

The transcription factor NF-E2 p45-related factor 2 (Nrf2) was regarded as one indispensable orchestrator to exert pleiotropic cytoprotective effects to maintain homeostasis, among which, antioxidant action was the primary function [70]. Under physiological conditions, Nrf2 combines with pressor protein Keap1, and is kept at a low level in cytoplasm usually. Upon high cellular stress, mitochondrial dysfunction, inflammatory stimulus, or others, Nrf2 is released from the complexes then translocated from the cytoplasm to the nucleus. As long as it is inside the nucleus, Nrf2 binds to *cis*-acting elements, namely antioxidant response elements (ARE), and activates downstream transcription and the expression of antioxidation-related genes, such as NADPH, NQO1, HO-1, etc. [71]. Many studies describe the activation of Nrf2-protected retinas in retinal diseases (Figure 3). Nrf2 displayed a protective effect to Müller cells when exposed to homocysteine (Hcy), one oxidative stress stimulator, and the depletion of Nrf2-induced retinal aging-related degeneration [72,73,74]. A recent study showed that Nrf2 interacted with sigma-1 receptor by a co-immunoprecipitation experiment in the cell line 661W. Analysis of electron microscopy immunogold labelling revealed that Nrf2 and sigma-1 receptors were co-localized in the cytoplasm, the nucleus, and the nuclear membrane via. The colocalization was also observed in mice retinal photoreceptor cells [37]. Meanwhile, the activation of sigma-1 receptor increased Nrf2-ARE binding, and Nrf2 expression in whole lysates, cytosolic, and nuclear fractions in 661W cells. In contrast, silencing of sigma-1 receptors reversed the expression of Nrf2 and potentiated cellular stress level [35,75]. In the Müller cells of sigma-1 receptor-absent mice, Nrf2 expression was decreased at the gene and protein level; additionally, the downstream antioxidant genes, such as Sod1, Catalase, Nqo1, Hmox1, Gstm6, and Gpx1 decreased, as well as their protein products. Furthermore, the ROS level was higher in cultured primary Müller cells in sigma-1 receptor knockout mice than that of the wild type mice [69]. *Pde6b^rd10^* mice is a type of inherited retinitis pigmentosa mice model in which rod and cone photoreceptors were lost resulting from the missense mutation in the phosphodiesterase 6β subunit in *rd10* mice. An in vivo investigation made it clear that sigma-1 receptor agonist administration was incapable of rescuing cone photoreceptor function in *rd10* mice when Nrf2 was absent. The amplitude of cone responses was decreased in *rd10/Nrf2*^−/−^ mice compared with that of *rd10* mice by ERG recording. Meanwhile, there was a larger cone photoreceptor cell loss in *Nrf2*-deficient mice [35]. Similar results were acquired in other tissues. In cultured primary hippocampal neurons, a gene array analysis showed that sigma-1 receptor silencing attenuated Nrf2 expression [76]. In the tissues of liver and lung, over expression of sigma-1 receptors compromised the upregulated oxidative stress level as well as ARE. The expression of downstream antioxidant genes was attenuated in sigma-1 receptor-deficient mice [77]. In cultured astrocytes, sigma-1 receptors performed anti-inflammatory and antioxidative effects via the Nrf2 signaling pathway [78]. However, there were different results in some studies. The expression levels of Nrf2-, Keap1-, and Nrf2-regulated antioxidant genes even increased in *rd10/Sig1R^−/−^* retina compared with *rd10* mice. In cultured primary cortical neuronal-glial cells, sigma-1 receptor deficiency promoted gliosis, and enhanced antioxidant capacity of cells under stressful conditions through Nrf2 signaling pathway [79,80]. Although the activation of Nrf2 plays a prominent role in retinal neuroprotective effects for sigma-1 receptors, the activation of Nrf2 alone did not produce the same effect as the activation of sigma-1 receptors [81].

### 3.2. MAPK Signaling Pathway

**Figure 4:**
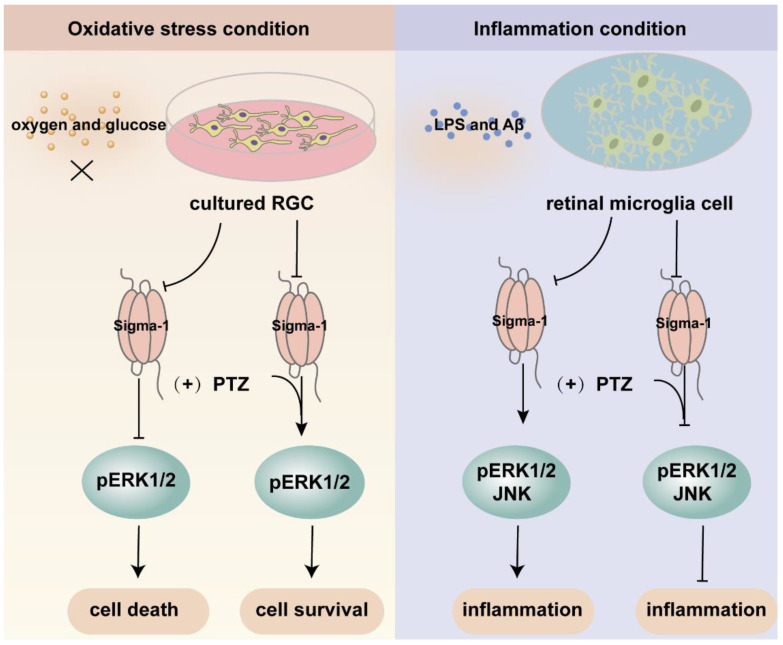
An overview of the mechanism of sigma-1 receptor modulates oxidative stress and inflammatory response in retina through MAPK signaling pathway. Upon oxygen and glucose deprivation in cultured RGC, the expression of sigma-1 receptor and pERK1/2 was suppressed. The activation of sigma-1 receptor enhanced the expression of pERK1/2 and cell survival. While under inflammation condition, the inflammatory response was suppressed through downregulating the expression of pERK1/2 and JNK by sigma-1 receptor.

Mitogen-activated protein kinases (MAPK) are three kinase signaling modules activated by multiple external and internal stimuli, and the pathways are composed of five subfamilies, the extracellular signal-regulated kinase (ERK1/2) pathway, the c-Jun kinase (JNK) pathway, the p38 pathway, the ERK3a/4 pathway, and the ERK5 pathway. Among them, ERK1/2 is the most thoroughly described and most extensively studied subset. ERK1/2 is widely expressed and activated by various inputs, leading to cellular proliferation, differentiation, survival, and cell cycle regulation [82,83,84]. Several investigations demonstrated that the ERK pathway was modulated by sigma-1 receptors (Figure 4). Within the retina, sigma-1 receptor activation enhanced the expression of phosphorylated ERK1/2 in NMDA-induced excitotoxicity retinal microglial cells, while it was diminished in sigma-1 receptor knockout mice [67]. ER stress was upregulated in the Müller cells of sigma-1 receptor knockout mice, and the mRNA and protein expression of phosphorylated ERK1/2 was decreased [52]. Likewise, a sigma-1 receptor agonist increased the expression of the phosphorylation of ERK1/2 and rescued cell death in primary RGCs suffering from oxygen glucose deprivation [44]. In FHL124, a defect of the sigma-1 receptor decreased pERK1/2, giving rise to growth inhibition and cell death [58]. Trabecular meshwork cells control the outflow of aqueous humor and maintain intraocular pressure balance. The dysfunction of trabecular meshwork cells leads to glaucoma and aberrant vision acuity. A study demonstrated that the activation of sigma-1 receptors protected cultured human trabecular meshwork cells from death via the InsR-MAPK ERK1/2 pathway under different air pressures [85]. Additionally, the activation of sigma-1 receptors promoted ERK phosphorylation and cognitive performance in brain injury mice models and Alzheimer’s disease (AD) model mice [86,87,88]. In light of this, the above studies suggested that the sigma-1 receptor played a pivotal role in neuroprotective effects by increasing the phosphorylation level of ERK1/2. However, under inflammatory conditions, pretreatment with the sigma-1 receptor agonist (+)-PTZ suppressed the phosphorylation of ERK1/2 and JNK, resulting in significant anti-inflammatory effects in LPS-induced inflammatory responses in retinal microglia cells [66]. Under oxidative stress, (+)-PTZ promoted ONHAs survival through the suppression of ERK1/2 phosphorylation, and the knockdown of sigma-1 receptors reversed the inhibition of pERK1/2 [54]. Based on the above two pieces of research, the sigma-1 receptor protected cellular function from inflammation and oxidative stress through the up-regulation or inhibition of the MAPK-ERK1/2 pathway. Some studies suggested that the sigma-1 receptor was also involved in the regulation of the JNK signaling pathway, which was activated by cellular stress, such as ROS, a hypertonic environment, and direct DNA damage [83]. Treatment with a sigma-1 receptor agonist decreased the elevated phosphorylation of JNK in rat retina suffering from Aβ-induced cytotoxicity and in retinal microglia under LPS-induced inflammatory responses [66,89].

### 3.3. Other Related Signaling Pathways

In addition to the two signaling pathways mentioned above, there were other related molecular mechanisms that might be also involved. The up-regulation of brain-derived neurotrophic factor (BDNF) was suggested to enhance the effect of antidepressants that blocked the reuptake of serotonin and norepinephrine to presynaptic terminals [46]. The BDNF-TrkB signaling pathway was also modulated by the sigma-1 receptor [90]. Mysona found that (+)-PTZ increased the expression of mature BDNF (mBDNF) protein in mouse retinas; in the meanwhile, the deletion of the sigma-1 receptor led to deficits of mBDNF in retinas. In vitro, BDNF secretion was significantly improved by (+)-PTZ treatment in ONHAs compared to the vehicle groups [91]. The activation of sigma-1 receptors modulated the function of several ligand-gated and voltage-gated ion channels, including NMDA- and AMPA-mediated cation channels, and calcium channels. The overstimulation of the ionotropic NMDA receptor led to an increase in intracellular calcium and cell death eventually. The death of retinal ganglion cells in diabetic retinopathy and glaucoma was thought to be mediated by excitotoxic damage through NMDA receptors [92]. (+) SKF10047 suppressed NMDA receptor-induced inward currents in rat retinal ganglion cells and the effect was blocked by a G-protein inhibitor. Further exploration suggested that the intracellular Ca^2+^-dependent PLC-PKC pathway was involved [63]. Research from Liu indicated that the activation of sigma-1 receptors inhibited AMPA receptor-mediated excitatory postsynaptic current by light stimulation in rat retinal ganglion cells, and the suppression was PKG pathway-dependent [64]. Another report revealed that the preincubation of a sigma-1 receptor agonist decreased influx of calcium through a direct interaction with an L-type voltage gated calcium channel in rat retinal ganglion cells [61].

## 4. Neuroprotective Effects of Sigma-1 Receptor

**Figure 5:**
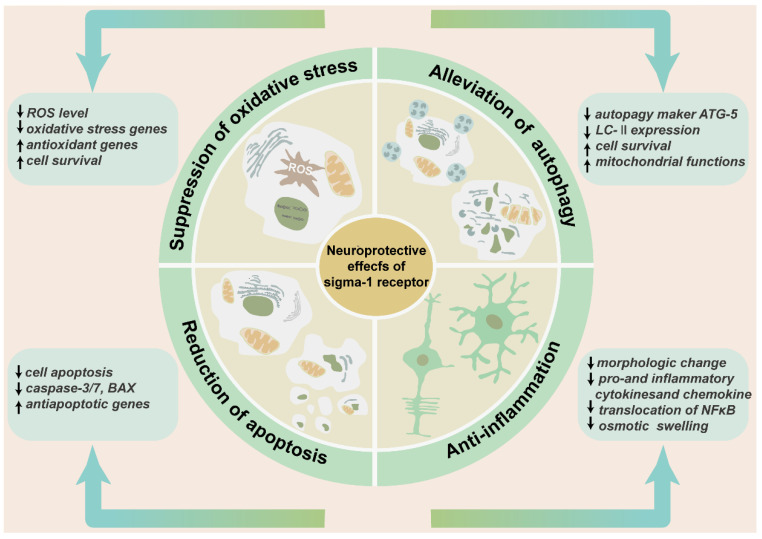
The neuroprotective effects of sigma-1 receptor in retina. The schematic diagram enumerates the protective effects of sigma-1 receptor in retina mainly including suppression of oxidative stress, alleviation of pathological autophagy, and reduction of cell apoptosis and anti-inflammation. These effects contribute to preserving retinal thickness and architecture, reducing production of inflammatory factors and inflammatory cells, and enhancing cell survival.

Many studies have confirmed the protective role of the sigma-1 receptor in multiple retinopathy models and a variety of cell subtypes in the retina. (+)-PTZ, SA4503, and neuroactive steroids were the most widely used agonists, PRE-084, (+) SKF10047, and (−)-MR22 are also involved. BD-1047, BD-1063, and NE-100 were the most common antagonists used to inhibit the physiological and biochemical effects of agonists (Table 1). Intravitreal injection of SA4503 rescued retinal impairment caused by exposure to excessive light. Morphology observation confirmed that AS4503 preserved retinal structure and prevented retina from thinning [36]. The treatment of neuroactive steroids attenuated the accumulation of lactate, increased the level of glucose and ATP, and preserved the thickness of the INL and IPL in a rat ischemia–reperfusion injury model. This protective function was almost equivalent to the administration of the selective agonist PRE-084. All the above effects were prevented by pre-treatment of antagonist BD-1047 [93,94]. Similarly, dehydroepiandrosterone (DHEA), one endogenous sigma-1 receptor agonist that was loaded by an intraocular RGC-targeted drug delivery system, reduced NMDA-induced neurotoxic effects to RGCs, and inhibited the activation and oxidative stress level of retinal microglia and macroglia [92]. Another research prompted that the deletion of the sigma-1 receptor exacerbated NMDA-induced RGCs death [54]. In *rd10* mice, (+)-PTZ improved retinal structure, reduced cone death, and rescued the cone function by ERG recording. In the meanwhile, (+)-PTZ weakened the gliosis of Müller cells, and the activation of microglial cells and oxidative stress [75,95]. In a chronic ocular hypertension rat model, pregnenolone not only reduced intraocular pressure and reversed RGC deaths, but also increased the thickness of the IPL [96]. In the diabetic retinopathy mouse model, the intraperitoneal injection of (+)-PTZ preserved the thicknesses of the INL and IPL and maintained radial fiber structure. Apoptosis and oxidative stress in retinal neurons were relieved in the GCL and INL [48]. Retinal edema, one of the developing processes of many ischemic and inflammatory retinopathy models, causes the swelling of Müller cells end feet in the nerve fiber layer [97]. Hypotonic solution perfusion induced an enlarged Müller cells volume. PRE-084 suppressed the swelling of Müller cells [65]. (+) SKF10047 suppressed NMDA inward current in retinal ganglion cells [63]. (−)-MR22 was investigated to relieve retinal degeneration in a rat ischemia–reperfusion injury model [98]. The sigma-1 receptor played a protective role mainly by mediating the oxidative stress level, reducing mitochondrial autophagy, and decreasing cell apoptosis and anti-inflammation [99] (Figure 5).

### 4.1. Suppression of Oxidative Stress

Physiologically, ROS are generated in an oxidation–reduction procedure, mediating proteins, lipids, and DNA metabolism. Disequilibrating oxidative stress between ROS and antioxidant molecules is one trigger of pathological processes and leads to the dysfunction of cell/tissue/organ [102,103]. Oxidative stress is tightly associated with a variety of retinal degenerative diseases, including diabetic retinopathy, glaucoma, age-related macular degeneration, and retinitis pigmentosa [104,105]. An action mechanism of sigma-1 receptors exerting neuroprotective effects was to regulate the level of oxidative stress in cells under pathological conditions [106,107]. At present, the majority of studies show that the sigma-1 receptor regulates oxidative stress and the expression of antioxidation-related genes. In rMC-1, NO and ROS treatment increased the binding activity of (+)-PTZ to sigma-1 receptor [51]. In primary ganglion cells and the 661W cell line, the activation of the sigma-1 receptor diminished the ROS-, cellular stress-, and oxidative stress-related genes, PERK, ATF6, IRE1, and CHOP. Additionally, the activation of the sigma-1 receptor improved the expression of the antioxidation-related genes, Nrf2, Nqo1, Catalase, and Hmox1 and preserved neurite projection [75,108]. In rat retinal microglia, (+)-PTZ reduced the LPS-induced intracellular ROS level and the NO release [66]. In vivo RGC-targeted DHEA downregulated the mRNA level of oxidative stress-related genes that were up-regulated by NMDA stimulation in wild type mice [92]. In ONHAs, (+)-PTZ treatment protected the cells from oxidative stress and improved cell survival [54]. However, the situation was different in sigma-1 receptor knockout mice. The expression of pro-oxidant factors, such as ROS, ER stress-associated genes, and Keap1, suppressor protein of Nrf2, were enhanced in Müller cells in sigma-1 receptor-deficient mice. On the contrary, the expression of antioxidant factors were down-regulated, such as Nrf2, antioxidant genes, and the glutamate–cystine exchange transporter xCT [52,69]. In the retina of *rd10* mice, it was found that a sigma-1 receptor agonist significantly reduced lipid and protein oxidation levels and upregulated the expression of the antioxidant gene Nrf2 and other antioxidant related genes [95]. Interestingly, different findings emerged in a follow-up study. In the cone cells of sigma-1 receptor knockout *rd10* mice, results showed higher expression levels of Nrf2 protein and mRNA andNrf2-regulated downstream genes. When the sigma-1 receptor was knocked-out, all the above-described factors that were facilitated by cell survival should be downregulated, but not be upregulated actually [79].

### 4.2. Alleviation of Pathological Autophagy

Autophagy, also named the programmed survival process of cells, is the universal and conserved degradation, and one re-using pathway of intracellular components and organelles. Pathologically, autophagy performs as a pivotal mediator of cell responses. Autophagy dysfunction is seriously responsible for protein homeostasis in cells and leads to the occurrence of many diseases [109]. Autophagy is highly correlated with several functions in retina, such as retinal development, cell corpse engulfment, degradation, neurogenesis, phototransduction, and retinal aging. Autophagy dysregulation leads to age-related macular degeneration, glaucoma, optic neuropathies, and photoreceptor degeneration [110,111].

In retina, the activation of sigma-1 receptors reduced pathological autophagy and increased cell survival. The inhibition of sigma-1 receptors increased the expression of autophagy marker ATG-5 during starvation in hungry RPE1 cells, which indicated a role of the sigma-1 receptor in autophagosome expansion [112]. In *rd10* mice, the absence of sigma-1 receptors enhanced the autophagy level through upregulating the expression of the autophagy-related protein LC3-II [41]. An in vitro cell line study showed that either the down-regulation of sigma-1 receptor or antagonist intervention suppressed the expression of programmed cell death ligand 1 (PD-L1), and increased tumor degradation via selective autophagy. The absence of sigma-1 receptor contributed to accumulation of autophagic vesicles [113,114,115]. In a stroke model, sigma-1 receptor increased cell survival by declining the autophagy of pericytes in the blood–brain barrier [116]. Mitochondria are key in the center of cellular metabolism, ATP production, and the generation of ROS. Mitophagy is an adequately studied type of cargo-specific autophagy. Damage to mitochondria causes engulfment into vesicles, eventually leading to mitophagy [117,118]. An investigation indicated that a missense mutation of sigma-1 receptor in familiar amyotrophic lateral sclerosis (ALS) patients resulted in declining mitochondria ATP synthesis and mitochondria impairment [119]. In the severe infection of SARS-CoV-2, sigma-1 receptor agonists activated mitophagy to suppress ER stress, preventing infection from damaged mitochondria, resulting in a decrease in hospitalization and death [120]. A change of mitochondrial membrane potential is one of the early responses in mitophagy. An agonist of sigma-1 receptors partially reversed the change of mitochondrial membrane potential in light—exposure 661W cells [36]. The pre-treatment with a sigma-1 receptor agonist or the overexpression of the sigma-1 receptor restored the decreased mitochondrial membrane potential and recovered mitochondrial functions in ganglion cells deprived of oxygen and glucose [100]. A recent paper demonstrated that the makers of lysosomal degradation VDAC1, TIM23, and autophagosome LC3-II were accumulated in a sigma-1 receptor knockout retinal explant. The deficiency of sigma-1 receptors affected the interaction with key membrane fusion proteins ATG14, STX17, and VAMP8, resulting in damage to the fusion function of autophagosomes and lysosomes and mitochondrial autophagy. The above case was rescued by the re-expression of the sigma-1 receptor. These findings not only revealed the relationships between the sigma-1 receptor and mitophagy, but also provided novel insights into understanding the possible regulatory mechanisms of mitochondrial autophagy damage in retina [121].

### 4.3. Reduction of Cell Apoptosis

Apoptosis is another fundamental common form of cell death that prevents the inflammatory response caused by the spillage of the intracellular components from dead cells [122]. Under pathological conditions, impaired or mutated retinal cells result in apoptosis, leading to a variety of retinal degenerative diseases [123]. Bcl-2 family proteins are very pivotal regulators in apoptosis pathways. Interactions between antiapoptotic protein, Bcl-2, and proapoptotic protein, Bax occurred in endoplasmic reticulum and mitochondria, contributing to the manipulation of intracellular calcium concentrations and energy metabolism, respectively [124]. It was reported that the sigma-1 receptor displayed a role in neuroprotection by modulating Bcl-2 in cultured primary cortical neurons [125]. In cultured primary retinal ganglion cells, (+)-PTZ decreased homocysteine-induced cytotoxic effects and facilitated cell survival [45]. In vitro studies indicated that caspase-3, Bax, and the level of cell apoptosis decreased via the sigma-1 receptor, in the meantime, the apoptotic inhibitor survivin increased [43,60,108]. In the 661W cone cell line, SA4503 treatment reduced light exposure-induced cell damage and the activation of caspase 3/7 [36]. In Aβ peptide-induced neurotoxicity rat retinas, PRE-084 produced neuroprotective effects by the reduced expression of pro-apoptotic cytokine tumor necrosis factors related to apoptosis, inducing ligand (TRAIL) and proapoptotic protein Bax [89]. In *Ins2^Akita/+^* diabetic mice, (+)-PTZ decreased the expression of proapoptotic genes Cflar and STKS and increased the expression of the antiapoptotic gene Eef1a2d [108]. In sigma-1 receptor deficient mice, TUNEL and ultrastructural analysis results showed that the apoptosis of retinal ganglion cells was characterized by the disappearance of the cell body, the destruction of optic nerve axons and the encompassment by phagocytes. mRNA levels of Bcl-2 began to decline at 6 weeks of age, and the downregulated expression level of Bcl-2 was more pronounced at 24-weeks-old and 52-weeks-old. In the meanwhile, the deletion of the sigma-1 receptor was accompanied with the downregulation of neuroprotective factors αB-crystallin, which was modulated by Bcl-2 [38,52,67,126].

### 4.4. Anti-Inflammation

Retinal diseases usually showed increased inflammatory responses. Microglia are the important population of immune cells in retina, and they secret inflammatory cytokines aggravating the related retinal diseases [127]. (+)-PTZ consumingly inhibited morphologic changes of microglia cells and suppressed the LPS-reduced release of inflammatory cytokines and chemokines, such as TNF-α, IL-10, and MCP-1. At the same time, (+)-PTZ lowered the phosphorylation of JNK and ERK, both of which were tightly involved in inflammatory reactions [66]. Müller cells are another primary type of participator in retinal inflammatory responses. In cultured primary Müller cells, the activation of the sigma-1 receptor blocked the secretion of specific proinflammatory cytokines MIP1γ, MIP2, MIP3α, and IL-12 accompanied with the declined expression of genes encoding the above pro-inflammatory cytokines. NFκB, a key transcription factor mediating inflammatory processes, was translocated from the cytoplasm to the nucleus to trigger the release of inflammatory cytokines by the inflammatory inducer-LPS. A study corroborated that the sigma-1 receptor was essential in NFκB mediating the inflammatory response. In *σR1^+/+^* Müller cells, (+)-PTZ reduced the translocation of NFκB, nevertheless, the transfer process of NFκB failed to be reversed in sigma-1 receptor knockout^−^ mice [128]. Hypoosmolality environments of retinal cells in inflammatory conditions causes the edema of retinal tissue, which probably leads to visual impairment or even blindness eventually [129]. Water accumulation in retinal neurons and glial cells is a pathogenic factor provoking retinal degeneration under ischemic hypoxic and inflammatory conditions. A previous study indicated that PRE-084 inhibited the osmotic swelling of rat Müller cells [65]. The neurotoxicity of NMDA increased the proliferation of microglial and Müller cells in retinas and further exacerbated inflammation, leading to the death of RGCs. DHEA reduced mRNA and protein expression of *iba1* and GFAP, markers of microglial and Müller cells, respectively [92]. By the same token, the activation effect of microglia and Müller glial was decreased by (+) PTZ treatment in *rd10* mice [95], whereas the activation of Müller cells was accelerated in *rd10* mice lacking the sigma-1 receptor [41].

## 5. The Sigma-1 Receptor-Associated Diseases

### 5.1. Diseases in Retina

With an increasing understanding of sigma-1 receptor, the more indispensable role it plays in physiological and pathological conditions. Defects of sigma-1 receptors cause a series of adverse consequences. All above studies implicate that the sigma-1 receptor may have the potential to be a therapeutic target in retinal diseases, such as glaucoma, retinitis pigmentosa, and diabetic retinopathy (Table 2).

Glaucoma is one retinal neurodegenerative disease leading to irreversible blindness. It is associated with an elevated intraocular pressure and accompanied with the death of retinal ganglion cells, mitochondrial dysfunction, and ER stress [99,160]. The sigma-1 receptor is involved in the modulation of intraocular pressure in the iris-ciliary body and trabecular cells [56,85]. In cultured primary retinal ganglion cells, the agonist of the sigma-1 receptor prevented the cell death from the intervention of cytotoxic substances (for example glutamate and, homocysteine) and nutritional deprivation. The agonist played a protective role by regulating apoptosis-associated proteins, Bax, and caspase-3 [43,44,45,60], and rescuing the decreased mitochondrial membrane potential [100]. The activation of the sigma-1 receptor exerted a protective effect in cell survival in an ischemia–reperfusion injury animal model by enhancing intraocular pressure [94,98]. The intraperitoneal administration of pregnenolone (PREG) prevented increased intraocular pressure in a rat glaucoma model induced by episcleral vein cauterization. mRNA and protein analysis indicated that PREG partially reversed the downregulation of sigma-1 receptors in retina tissues, preserved the thickness of the inner plexiform layer, and reduced the cell loss of ganglion cells [96]. A more potent and straightforward method to investigate functions of the sigma-1 receptor in retinal diseases was the use of sigma-1 receptor knockout mice. In an optic nerve crush model, the loss of retinal ganglion cell was increased in sigma-1 receptor-deficient mice compared with wild-type mice [39]. The gene deletion of the sigma-1 receptor caused a late-onset dysfunction in retinas. It was mainly manifested by a marked decrease in b-wave amplitudes in electroretinogram examinations, weakened scotopic vison responses, severe retinal ganglion cells loss, axonal breakage of the optic nerve head, obviously intracellular mitochondrial swelling, and increased apoptosis of microglial cells until the age of 59 weeks [38]. Another piece of research indicated the increased intraocular pressure in sigma-1 receptor-deficient mice [132]. The subsequent exploration showed no significant differences in ER stress-relevant genes in the entire retinas of sigma-1 receptor knockout mice; however, the mRNA expression of the antioxidant gene Bcl2 was severely reduced from postnatal 4 days to 52 weeks. The expression level of NFκB and phosphorylated ERK presented a downregulated trend. It was postulated that the neuroprotective role of the sigma-1 receptor in glaucoma was probably to regulate the anti-apoptotic pathway [52]. Intraperitoneal injection of (+)-PTZ ameliorated RGC death induced by the neurotoxicity of NMDA, while sigma-1 receptor-deficient suppressed the protective effect of (+)-PTZ [67]. DHEA was one endogenous ligand of the sigma-1 receptor. In an RGC-targeted intraocular delivery system, DHEA displayed a predominant anti-apoptosis effect, an anti-inflammatory response, and antioxidant stress to the RGC layer when the cells were damaged by NMDA. The above in vivo study implied the therapeutic role of the sigma-1 receptor in glaucoma [92]. BDNF was a vital protective factor to support RGC function and the optic nerve head was the axon of the RGC leaving in retina. (+)-PTZ increased the mBDNF protein level in the optic nerve head [91]. (+)-PTZ treatment increased the cell viability of ONHAs, as an essential glia to support the function of the RGC, and attenuated oxidative stress dependently when exposed to oxidative stimulus factors [54,161].

Diabetic retinopathy leads to severe visual impairment and even blindness as a complication of diabetes. It is often accompanied with retinal microvascular abnormalities, retina-vessel barrier impairment, the occurrence of inflammation, the thinning of retinal thickness, the loss of RGC reducing the reaction in electroretinography detection, and increased endoplasmic stress [104,162]. Early investigation manifested the similar mRNA and protein expression pattern of the sigma-1 receptor in cultured retinal ganglion cells under hyperglycemic conditions, retina in streptozotocin-induced diabetic mice, and wild type mice [47]. In the *Ins2*^Akita/+^ diabetic retinopathy mice model, intraperitoneal injection of (+)-PTZ at the onset of diabetes for 22 weeks maintained retinal structural integrity, increased thickness of mouse retina, especially the IPL and INL, and rescued the cell number of the ganglion cell layer compared with the untreated groups. At the same time, (+)-PTZ treatment preserved the radial morphology of Müller cells, reduced the accumulation of reactive oxygen and reactive nitrogen species [48], and decreased ER stress-related genes, such as PERK, ATF6, IRE1, and ATF4 [108]. The result of DNA microarray analysis in *Ins2^Akita/+^* mice indicated that (+)-PTZ injection reversed the expression of 23 genes that were associated with apoptosis, oxidative stress, axon outgrowth, calcium binding, and cell differentiation among 29992 genes [108]. Even the delayed injection of (+)-PTZ in *Ins2^Akita/+^* mice at 4 weeks or 8 weeks after the onset of diabetes decreased cell loss of GCL and gliosis of Müller cells and preserved the neatly nuclear layer and retinal architecture compared with no-treatment *Ins2^Akita/+^* mice. Fundoscopy indicated vitreal opacities and retinal vessel leakage in sigma-1 receptor deficient *Ins2^Akita/+^* mice at the age of 12 weeks or 16 weeks. Furthermore, retinal morphology was more fragmented when the sigma-1 receptor was absent in *Ins2^Akita/+^* mice than that of *Ins2^Akita/+^* mice, which suggested that a defect of the sigma-1 receptor accelerated the pathological features of diabetic mice [49]. Although a typical pathological phenomenon, such as retinal ischemic injury, blood leak phenomenon, and abnormal intraocular pressure, did not emerge in mice lacking the sigma-1 receptor at ages from 5 to 59 weeks, there was a decrease b-wave amplitude to scotopic threshold response until 12 months [38]. The protection was also weakened under stress leading by optic nerve crush in sigma-1 receptor deletion mice [39]. Unlike the previous study reviewed, it presented increased intraocular pressure and blood glucose and insulin levels in a streptozotocin-induced diabetic model in sigma-1 receptor knockout mice. Similarly, the cell loss of RGC was enhanced in sigma-1 receptor-deficient mice when diabetics occurred [132].

Retinitis pigmentosa (RP) is a one class inherited retinal progressive degenerative disease characterized by abnormalities of the retinal pigment epithelium and impairment of photoreceptors, ultimately resulting in vison loss. Currently, one strategy for RP therapy is to acquire neuroprotection to maintain retinal architecture integrity [130]. As mentioned previously, both in vitro and in vivo studies showed that sigma-1 receptors were expressed in photoreceptor cell lines and photoreceptor cells and played a pivotal role to protect photoreceptor cells from damage. In vitro pharmacological studies reported the expression of sigma-1 receptors in 661W. SA4503 reduced the light exposure-induced injury to 661W cells, up-regulated expression of the sigma-1 receptor and diminished the level of oxidative stress and expression of antioxidant-relevant genes when cells were under oxidative stress [36,37]. When mice were exposed to a strong light stimulus, the damage to the retina was attenuated by the intravitreal injection of SA4503. The amplitudes of a-waves and b-waves were increased, as well as the thickness, especially the ONL with SA4503 treatment [75]. In vivo study revealed that the intraperitoneal administration of (+)-PTZ for several weeks at p14 ameliorated cone function. ERG recording indicated enhanced photopic responses at p35, which was accompanied with decreased cone nucleus loss by immunofluorescence staining. Furthermore, whole retinal thickness and the detachment of photoreceptor cells were preserved at p42 and p21 after (+)-PTZ injection. The results of histomorphology analysis presented less photoreceptor cell nucleus loss in the (+)-PTZ-*rd10* group at p42. Meanwhile, (+) PTZ treatment performed as a “protective molecule” in other processes, including the loss of GCL, the reactive gliosis of microglial and Müller cells, and the oxidative stress level of lipids and proteins. While in sigma-1 receptor-deficient *rd10* mice, the above protective effects to cone cells were reversed, suggesting the essential role of the sigma-1 receptor in retinitis pigmentosa [95]. Further investigation indicated that the early intervention of sigma-1 receptors with (+)- PTZ at p14 in *rd10* mice presented a more profound protection to photoreceptors than treatment at p18, p21, and p24. Visual acuity by an optokinetic tracking system recoding, the amplitude of electroretinograms, and the retinal structure all performed better in the (+)-PTZ-*rd10* group [81,101]. When compared with two other agonists, SA4503 and PRE-084, only (+)-PTZ presented in vivo protection in *rd10* mice [75]. While in sigma-1 receptor knockout *rd10* mice, the above results were suppressed. The subsequent exploration suggested that the antioxidant signaling pathway Nrf2 was involved in the neuroprotection of the sigma-1 receptor to photoreceptor cells [35]. Sigma-1 receptor deletion exacerbated the loss of entire retinal cells, accompanied with the significant reduction of photopic ERG responses. In sigma-1 receptor knockout *rd10* mice, the loss of rod photoreceptor cells occurred before 5 weeks of age, while the loss of cones was exacerbated after being 6 weeks old. Similarly, gliosis was accelerated in Müller cells with an increased expression of GFAP, oxidative and ER stress associated signal molecular increased. For example, the expression of the apoptotic protease caspase3, the autophagy marker LC3-II, and the ER stress marker CHOP, was markedly upregulated at the early stage in sigma-1 receptor knockout *rd10* mice [41,131].

### 5.2. Other Diseases

Apart from the above retinal diseases laid out here, a series of studies indicated that the sigma-1 receptor was one potential therapeutic target for several other diseases including neurodegenerative diseases, chronic pain, cancer, drug addiction, and cardiovascular disease. The phenotype and progression of neurodegenerative disorders, consisting of Huntington disease [133,163], Alzheimer’s disease [134,135,136], amyotrophic lateral sclerosis [137,138,139], and Parkinson’s disease [140,141,142], were exacerbated when the sigma-1 receptor was blocked [163]. The sigma-1 receptor was also a promising therapeutic target for the treatment of alcohol addiction [143], methamphetamine addiction [144,145,146], and cocaine addiction [147,148,152]. The sigma-1 receptor antagonist showed analgesic effects to relieve the symptoms in several peripheral neuropathy models [153,154,155,156]. MR309, a selective and novel antagonist of the sigma-1 receptor [164], has been tested in a phase II clinical trial, suggesting the promising action for oxaliplatin-induced peripheral neuropathic pain relief in patients [165]]. A good deal of evidence supported that the sigma-1 receptor was a promising target for cancer treatment although there was no clinical drugs application. The upregulated expression of sigma-1 receptor protein was detected in cancer cell lines [166,167] and several tumors such as prostate cancer [149], esophageal squamous cell carcinoma (ESCC) [150], and hilar cholangiocarcinoma [151]. It also presented cardio protection in cardiovascular disease [157,158,159].

## 6. Conclusions and Perspectives

Since the original discovery of the sigma-1 receptor, multiple investigations have provided insight into the potential protective role of the sigma-1 receptor in the retina through oxidative stress, cell autophagy, cell apoptosis, and anti-inflammation. Synthetic and endogenous agonists and sigma-1 receptor-deficient mice were applied to identify the molecular mechanisms and signaling pathways in the retina. The reviewed pieces of research suggest the promising therapeutic role of the sigma-1 receptor in retinal degenerative diseases, especially glaucoma, diabetic retinopathy, and retinitis pigmentosa. Nevertheless, as one widespread distributed molecular chaperone, the more specific molecular mechanism of neuroprotective effects played by the sigma-1 receptor in retinal degenerative diseases remains to be deeply explored and further studied. For example, the conditional knockout of sigma-1 receptors in different retinal developing stages and distinct subtypes of retinal neurons.

## Figures and Tables

**Table 1:** Ligands of sigma-1 receptor in retinal pharmacological researches.

Ligand	Activity	In Virto/In Vivo	Function	Reference
(+)-PTZ	Agonist	rMC-1, RGC	Decrease cell death, ER stress Preserve neurite projection Restore mitochondrial function Preserve retinal architecture Reduce reactive gliosis Enhance visual acuity, ERG response	[38,48,49,54,81,95,100,101]
SA4503	Agonist	661W, Retina	Alleviate NMDA-induced neurotoxic and light exposure-induced cell damage Decrease expression of caspase 3/7 Prevent retina from detachment Auxiliary diagnostic reagent for retinal diseases	[36,57,59,75]
Neuroactive steroids	Agonist	Retina	Preserve thickness of INL, IPL Decrease intraocular pressure Attenuate accumulation of lactate Reduce cell loss of RGC	[54,93,94,95]
PRE-084	Agonist	661W, Retina	Improve cell viability Decrease oxidative stress Suppress osmotic swelling of retina Reduce expression of Bax and JNK Preserve retinal thickness from ischemia injury	[75,89,94]
(+) SKF10047	Agonist	RGC-5, Retina	Reduce activation of caspase3 and Bax Increase cell viability Suppress NMDA and AMPA-induced inward current Inhibit calcium ion influx through L-type VGCC	[60,62,63,64]
(−)-MR22	Agonist	Retina	Alleviate retinal ischemia-reperfusion damage	[98]
BD-1047	Antagonist	RGC	Reverse the neuroprotection of SA4503 Block the effect of SKF10047 to NMDA-mediated eEPSC of GCs	[36,89]
NE-100	Antagonist	APRE-19	Suppress the neuroprotective effect of agonist to DNA damage	[98]
BD-1063	Antagonist	Microglia	Eliminate the anti-inflammation of sigma-1 receptor agonist	[67]

**Table 2:** Sigma-1 receptor relevant disorders.

Disorders	Study Model	Reference
Retinal degenerative diseases		
Glaucoma	Cultured primary retinal ganglion cell *SigR*^−/−^ mice Ischemia-reperfusion injury animal NMDA-induced animal model	[38,39,43,44,45,52,54,61,67,91,92,94,96,99,100]
Retinitis pigmentosa	661W cell line, Wild type mice, *Rd10* mice, *Rd10/SigR*^−/−^ mice	[35,36,37,41,68,75,81,96,101,130,131]
Diabetic retinopathy	Cultured primary retinal ganglion cell *Ins2^Akita/+^, Ins2^Akita/+^ /Sig1R^−/−^* and streptozotocin induced Diabetic retinopathy mice model	[38,39,47,48,108,132]
Neurodegenerative diseases		
Huntington disease	Mutant Huntingtin transfected mice, *SigR^−/^*^−^ mice	[133]
Alzheimer’s disease	Mouse model of Alzheimer’s disease AD patients	[134,135,136]
Amyotrophic lateral sclerosis	Mouse model of ALS disease Motor neuron-like cell line model (NSC34)	[137,138,139]
Parkinson’s disease	Parkinson’s disease patients MPTP-*SigR^−/^*^−^ mice and MPTP-WT mice	[140,141,142]
Addiction		
Alcohol addiction	WT and Sig-1R knockout mice	[143]
Methamphetamine addiction	WT and Sig-1R knockout mice	[144,145,146]
Cocaine addiction	HEK293T cell and WT mice	[147,148]
Cancer		
Prostate cancer	LNCaP, VCap,22RV1, PC3, C4-2 and LAPC4	[149]
Esophageal squamous cell carcinoma	Human ESCC cell lines KYSE180, KYSE150, and EC109	[150]
Hilar cholangiocarcinoma	Tissues from hilar cholangiocarcinoma patients	[151]
Others		
Peripheral neuropathic pain	Mouse model of osteoarthritis pain CFA-induced inflammatory model Chronic nerve constriction injury (CCI)model Acute and chronic oxaliplatin-induced peripheral pain	[152,153,154,155,156]
Cardiovascular disease	Ovariectomized female rats	[157,158,159]
